# Transient receptor potential vanilloid 3 activation accelerates keratinocyte migration in vitro but not dermal wound healing in vivo

**DOI:** 10.1016/j.molpha.2025.100084

**Published:** 2025-10-27

**Authors:** Carolin Zosel, Anne-Kathrin Krause, Anne Müglitz, Yan-Qin Zuo, Ute Krügel, Michael Schaefer

**Affiliations:** Rudolf-Boehm-Institute for Pharmacology and Toxicology, Leipzig University, Leipzig, Germany

**Keywords:** Transient receptor potential vanilloid 3 channel, Skin wound healing, Cell migration and proliferation, Epidermal growth factor receptor, Phosphoinositide 3-kinase

## Abstract

The non-selective Ca^2+^ permeable transient receptor potential vanilloid 3 (TRPV3) ion channel is highly expressed in mouse keratinocytes, where its activation causes an accelerated cell migration and proliferation via an epidermal growth factor receptor–mediated mechanism in vitro. Therefore, TRPV3 has been proposed as a potential target to accelerate dermal wound healing. In this study, we provide an insight into the effects of TRPV3 activation on mouse keratinocytes and skin wound healing in vitro and in vivo using the newly identified activator of TRPV3 1 (AV3-1). To investigate a possible TRPV3-mediated effect on dermal wound closure in vivo, 2 circular wounds were excised on the back of wild-type and TRPV3 knockout mice and topically treated with AV3-1 or the corresponding vehicle. Unexpectedly, the comparison of neither the wound areas nor the histologic parameters yielded a statistically significant difference between wild-type and TRPV3 knockout wounds. Supporting this notion, AV3-1 treatment could not induce any further acceleration of the wound closure compared to vehicle-treated wounds. Therefore, the described TRPV3-mediated acceleration of keratinocyte migration and proliferation in vitro cannot be directly translated into an in vivo context.

**Significance Statement:**

We here show that deficiency of the transient receptor potential vanilloid 3 (TRPV3) channel impairs mouse keratinocyte migration in vitro. In vivo, however, neither TRPV3 deficiency nor TRPV3 activation by the novel activator of TRPV3 activator 1 (AV3-1) had statistically significant effects on healing rates or reepithelialization of skin wounds.

## Graphical abstract

**Figure:**
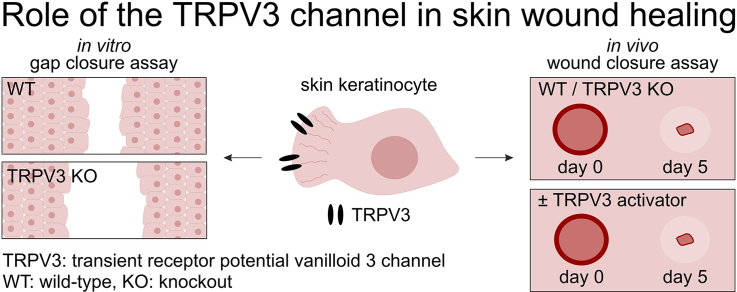

## 1 Introduction

Dermal wound healing consists of partially overlapping phases of inflammation, proliferation, and remodeling.^1^ The inflammatory phase is characterized by hemostasis and invading immune cells such as neutrophils and macrophages, clearing the wound bed from foreign particles and pathogens.^2^ Activated macrophages and platelets secrete transforming growth factor *α*, epidermal growth factor (EGF), and platelet-derived growth factor, inducing the proliferation phase.^3^ This second phase includes angiogenesis, the formation of granulation tissue by fibroblasts, and the beginning of reepithelialization by migrating and proliferating keratinocytes. In the remodeling phase, new epithelium is formed, and the collagen is reorganized with potential subsequent scar formation.^4^ On the one hand, a single disturbance in the process may lead to manifested wound healing defects because of the functional intertwining of the phases. On the other hand, the high interactivity of the signaling cascades in skin wound healing may give room to a relevant number of potential compensatory mechanisms, if one of the pathways should fail.

The thermosensitive Ca^2+^ permeable transient receptor potential vanilloid 3 (TRPV3) channel is functionally expressed in mouse keratinocytes^5^^,^^6^ and begins to open at temperatures of 33–35 °C.^7^ TRPV3 can be selectively stimulated with the newly identified, low-toxic activator of TRPV3 1 (AV3-1) that is described and validated in J. Janenz et al (manuscript in preparation). The TRPV3 channel has been described to form a signaling complex with the EGF receptor (EGFR),^8^ leading to EGFR activation by TRPV3 opening. The corresponding mechanism is thought to work via Ca^2+^ influx through TRPV3, activating Ca^2+^/calmodulin-dependent protein kinase II (CaMKII) and subsequent transforming growth factor *α* (TGFα) shedding, stimulating EGFR and nuclear factor *κ*-light-chain-enhancer of activated B cells (NF-κB) as downstream effectors.^9^ The mitogenic downstream signaling cascade of EGFR is known to induce cell proliferation and migration.^10^^,^^11^ Therefore, it is plausible that TRPV3 activation mediates an increased mouse keratinocyte migration and proliferation in vitro in an EGFR-dependent manner.^6^^,^^9^ Furthermore, TRPV3 opening enhances the vascular endothelial growth factor signaling in non–small cell lung cancer cells,^12^ suggesting a promoting role of TRPV3 in angiogenesis.

Taking the in vitro results to TRPV3’s role in keratinocyte migration and proliferation together, an accelerating effect of TRPV3 activity on dermal wound healing seems possible.^13^ Hence, corresponding in vivo studies are of particular interest. However, the existing in vivo studies provide contrary results. As expected, Aijima et al^14^ demonstrated a slower wound healing of the oral epithelium in TRPV3 knockout (TRPV3 KO) versus wild-type (WT) mice. In contrast, Miyamoto et al^15^ did not observe any statistically significant difference in the healing of excisional skin wounds in WT and TRPV3 KO mice. Both studies were limited to the comparison of untreated wounds in WT and TRPV3 KO mice because selective, non-toxic TRPV3 activators have not yet been available up to this time. This exploratory study aims to fill this gap by performing in vitro and in vivo wound healing assays with the new pharmacological TRPV3 activator AV3-1 to further elucidate TRPV3’s role in dermal wound healing.

## 2 Materials and methods

### 2.1 Reagents

All chemicals were purchased from Merck KGaA, unless stated otherwise.

### 2.2 Isolation of primary keratinocytes

Primary keratinocytes were isolated from neonatal mouse skin according to Li et al.^16^ Briefly, newborn (P0-P2) C57BL/6J WT or TRPV3 KO mice of both sexes were euthanized by decapitation. Limbs and tail were removed, and the skin was incised above the dorsal midline in a caudal-cranial direction. The dermal tissue was carefully detached from the body and digested for 12–18 hours in a solution containing 1 mg/mL collagenase/dispase in keratinocyte growth medium consisting of KBM Gold Keratinocyte Growth Basal Medium, calcium and phenol red-free supplemented with hydrocortisone, transferrin, epinephrine, gentamicin sulfate and amphotericin (GA-1000), bovine pituitary extract, human EGF, insulin (medium and supplements from Lonza), and 0.06 mM CaCl_2_. Following digestion, the epidermis was separated from the dermis, and the basal layer was treated with 0.025% trypsin for 20 minutes. Both basal and suprabasal keratinocytes were isolated from the epidermis and cultured on collagen A–coated coverslips or in 24-well plates in keratinocyte growth medium at 37 °C and 5% CO_2_ in a humidified atmosphere. Collagen-A coating was performed 24 hours prior to seeding, by incubating glass coverslips for 30 minutes at 37 °C with a 1:1 mixture of collagen A and PBS (pH = 3.2) and washing twice with PBS (pH = 7.4).

### 2.3 Transient transfection

Primary keratinocytes were seeded onto collagen A–coated glass coverslips and after 24 hours transfected with either 4 *μ*L LipofectAMINE 2000, 4 *μ*L LipofectAMINE 3000 (Thermo Fisher Scientific), or 4 *μ*L jetPEI (Polyplus) and 1 *μ*g of plasmid cDNA encoding the fusion protein of yellow fluorescent protein and the pleckstrin homology domain of the general receptor for phosphoinositides isoform 1 (YFP-GRP1(PH)). Imaging was performed 24 hours after transfection.

### 2.4 In vitro gap closure

Following isolation, primary keratinocytes were seeded into 2-well culture inserts (ibidi GmbH) placed in a 24-well plate (Sarstedt) and cultured for 48 hours at 37 °C and 5% CO_2_ in a humidified atmosphere. After formation of confluent monolayers in each well, inserts were removed to create a defined 500-*μ*m gap. Cells were washed once with PBS, and following gap closure was monitored every 6 hours with a label-free, non-perturbing system for live-cell imaging (Incucyte SX5, 4× objective, Sartorius) in keratinocyte growth medium at 37 °C, 5% CO_2_ and in a humidified atmosphere. Gap regions with no confluent monolayers at their boundaries were excluded from analysis. Gap areas were analyzed over time using ImageJ.^17^

### 2.5 Total internal reflection fluorescence microscopy

Primary keratinocytes seeded onto collagen A–coated coverslips were visualized with light-emitting diode total internal reflection fluorescence microscopy (LED-TIRFM), as previously described by Kogel et al,^18^ equipped with a quantitative complementary metal–oxide–semiconductor (qCMOS) camera (Orca Quest, Hamamatsu Photonics). Cells were kept in HEPES-buffered solution (HBS), containing 132 mM NaCl, 6 mM KCl, 10 mM HEPES, 1 mM MgCl_2_ (all from Carl Roth), 5 mM glucose (Th. Geyer), and 1 mM CaCl_2_ during imaging, unless stated otherwise. To induce Ca^2+^ influx, AV3-1 in HBS was added during TIRFM imaging.

### 2.6 TIRF microscopy imaging of subplasmalemmal Ca^2+^ sparklets

Primary keratinocytes were incubated in HBS supplemented with 10 *μ*M of the low-affinity Ca^2+^ indicator Cal520ff/AM (K_D_ = 9.8 *μ*M, Biomol), 1 mM probenecid (MedChemExpress LLC), and 0.1% bovine serum albumin for 30 minutes at 37 °C. After indicator loading, cells were washed with HBS and maintained in HBS with 2 mM CaCl_2_ during fluorescence imaging. The LED-TIRFM images were acquired with a frequency of 20 Hz, an exposure time of 50 ms, 2 × 2 binning, an excitation wavelength of 475 nm, and 25% of the maximum excitation intensity. As a light source, a Spectra III light engine (Lumencor) equipped with a 3 mm liquid light guide was used.^19^ To evoke subplasmalemmal Ca^2+^ concentration [Ca^2+^]_i_ fluctuations (Ca^2+^ sparklets), a final concentration of 3 µM AV3-1 was added.

### 2.7 TIRF microscopy imaging of transfected cells

Primary keratinocytes transfected with a cDNA plasmid encoding YFP-GRP1(PH) were imaged with an acquisition frequency of 0.1 Hz, an exposure time of 200 ms, 2 × 2 binning, an excitation wavelength of 510 nm, and 1% of the maximum excitation intensity. In a second set of experiments, EGFRs were inhibited by the addition of 100 nM N-(3-chlorophenyl)-6,7-dimethoxy-4-quinazolinamine (AG 1478) prior to imaging. To induce fluorescence signals, a final concentration of 15 *μ*M AV3-1 was applied during the measurements.

### 2.8 Fluorometric imaging plate reader assay

Serum from healthy adult human donors was obtained by coagulation of venous blood in a clotting activator gel (S-Monovette Serum Gel-CAT). AV3-1 (10 mM) was added to the serum and either freshly frozen (indicated as 0-hour incubation time) or incubated at 34 °C for 6 or 24 hours and then frozen. The ability to activate TRPV3 was assessed in a fluorometric imaging plate reader multiwell Ca^2+^ assay performed in a robotic liquid handling station equipped with a 96-tip multichannel head (Freedom Evo 150, Tecan) and an integrated home-built fluorescence plate imager as described earlier.^20^ The fluorescence of Fluo-4-loaded (4 *μ*M; Thermo Fisher Scientific) human embryonic kidney (HEK) cells expressing mouse TRPV3-CFP was monitored during acute application of AV3-1-free serum and AV3-1-containing samples at a final dilution of 1:1000 in HBS. Images were acquired with a 400-ms exposure time and a frequency of 2.5 Hz.

### 2.9 Animals

Adult C57BL/6J WT (The Jackson Laboratory, RRID: MSR_JAX:000664) and genetically matching TRPV3 KO (B6.129-*Trpv3*^*tm1Apat*^/J, RRID:IMSR_JAX:010773) mice were housed in groups of 2–3 animals under approved standard conditions of a 12-hour light–dark regime and access to food and water ad libitum. Four female and 17 male animals between 8–10 weeks old were used in the study. The experimental procedures were approved by the Institutional Animal Care and Use Committee (Animal Welfare Office Saxony; license no.: 25-5131/556/22) according to the German regulations for the welfare of laboratory animals. Every effort was made to minimize the stress and overall number of animals used in this study.

### 2.10 In vivo skin wound healing

To evaluate the in vivo dermal wound closure, two 5-mm biopsy punch wounds were excised on the back of C57BL/6J WT and TRPV3 KO mice (Supplemental Fig. 1). One day prior to the surgical procedure, the mice received tramadol (0.2 mg/mL) in sweetened drinking water as an analgesic. The dorsal fur was removed by shaving and application of depilatory cream (Balea).

One hour before the dermal biopsy, metamizol (s.c., 200 mg/kg) was administered. The animals were anesthetized with isoflurane (3% in O_2_) and placed on a heating mat. The skin was cleansed with 70% ethanol, and a skin fold was formed along the dorsal midline. A biopsy punch (diameter: 5 mm; Dieckhoff & Ratschow Praxisdienst GmbH & Co KG) was inserted through the fold to create symmetrical wounds on both sides of the midline. On days 1–4 postsurgery, one of the wounds was randomly chosen and topically treated every 24 hours with either 10 *μ*L of AV3-1 (100 *μ*M) in PBS or with 10 *μ*L of a combination of AV3-1 (100 *μ*M) with EGF (10 ng/mL) in PBS, whereas the contralateral wound received 10 *μ*L of the corresponding vehicle. The treatment solutions were administered by pipette under anesthesia. AV3-1 dilutions were prepared from a stock solution of 100 mM in DMSO (Th. Geyer). The administered doses of AV3-1 and EGF were 408.5 and 0.1 ng per day, respectively. After surgery, the mice received 0.4 mg/mL tramadol in sweetened drinking water for 48 hours. Photographs of the wounds and of an enclosed scale were taken with an RGB camera (*α*-5100, Sony) equipped with a Zoom Lens 1:4 objective (Canon), immediately after surgery and subsequently every 24 hours for 5 consecutive days under anesthesia. The wound areas were analyzed using ImageJ.

### 2.11 Histologic analysis

Five days postsurgery, mice were euthanized, and the whole wound tissue was explanted as a 1 × 0.5 cm skin sheet. Wounded tissue was fixed for 24 hours by floating in 4% paraformaldehyde in PBS at 8 °C and subsequently dehydrated in a TP 1020 tissue processor (Leica) according to the following protocol: 30% ethanol (5 minutes, Carl Roth), 50% ethanol (5 minutes), 70% ethanol (2 hours), 80% ethanol (4 hours), 100% ethanol (8 hours), 100% isopropanol (2 × 1 hours, Carl Roth), 100% n-butyl acetate (2 × 1 hours, Carl Roth), and 100% paraffin (2 + 2 × 1.5 hours, 60 °C, Carl Roth). Twenty-four hours after fixation, the tissue was embedded in paraffin using an EG1150 modular tissue embedding center (Leica). The paraffin block was then cooled down for at least 24 hours at 8 °C before the embedded tissue was sectioned into 7-*μ*m thin slices with a Jung Biocut 2035 microtome (Leica) and mounted onto poly-(l-lysine)-coated glass slides (Epredia). The slides were kept at 37 °C for 24 hours before being heated up to 60 °C for 3 hours and subsequently rehydrated with NeoClear (2 × 10 minutes, Carl Roth), 100% ethanol (2 × 10 minutes), 96% ethanol (2 × 5 minutes), 70% ethanol (2 × 5 minutes), and distilled water (1 × 5 minutes). Following rehydration, slices were RGB stained according to Serrano-Garrido et al,^21^ using solutions of Alcian Blue, fast green, and Sirius red. Briefly, the tissue was first stained with 1% Alcian Blue to visualize proteoglycans. Subsequently, the wounds were stained with 0.04% fast green and 0.1% Sirius red to stain keratins and collagen, respectively. Images were captured the following day with a transmission light microscope (BZ-800X, Keyence). The pictures were subsequently stitched together using ImageJ,^22^ and the wound and epidermal areas and lengths were analyzed. The epidermal area and length ratios were calculated by the following formulas:

(1) $$Epidermal\;area\;ratio=\frac{Epidermal\;area}{Wound\;area}$$

(2) $$Epidermal\;length\;ratio=\frac{Epidermal\;length}{Wound\;length}$$

The analyzing investigator was not blinded to group allocation.

### 2.12 Data and statistical analysis

Normal distribution of the data was tested using the Shapiro-Wilk test. Statistical significance (*P* ≤ .05) was tested using the *t* test for parametric data pairs and the Wilcoxon signed-rank test for non-parametric pairs. Multiple comparisons were performed with the two-way ANOVA with repeated measurements for parametric data or the Kruskal-Wallis ANOVA and the Dunn test for non-parametric data. All data are presented as box plots with interquartile ranges, and raw fluorescence data were analyzed with ImageJ. Calculated *P* values can only be interpreted as descriptive because of the exploratory nature of the study.

## 3 Results

### 3.1 Keratinocytes isolated from WT mice perform gap closure faster than cells from TRPV3 KO mice

**Fig. 1:**
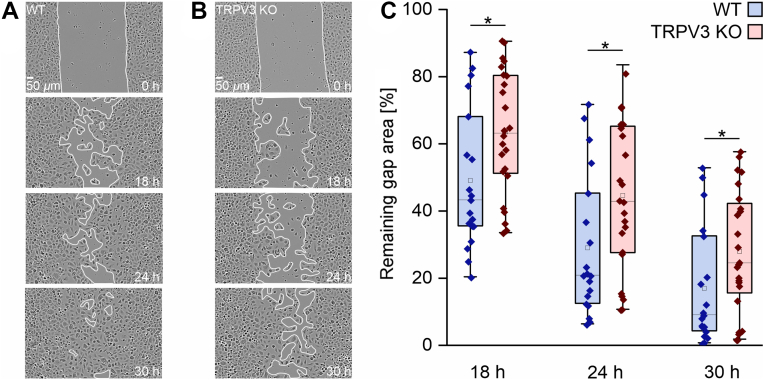
WT keratinocytes close gaps statistically significantly faster than TRPV3 KO keratinocytes in vitro. (A, B) Primary keratinocytes from WT (A) and TRPV3 KO (B) mice closing a 500-*μ*m gap (start at t = 0 hours) after 18, 24, and 30 hours of migration and proliferation. (C) Statistical analysis of the remaining gap areas as shown in (A) and (B) of WT (blue boxes) and TRPV3 KO (red boxes) keratinocytes after 18, 24, and 30 hours. Cells were kept in keratinocyte growth medium at 37 °C and 5% CO_2_ in a humidified atmosphere (n_WT_ = 12, n_TRPV3 KO_ = 13, ∗*P* ≤ .05, data not normally distributed).

Previously, Maier et al^6^ described an accelerated migration of immortal mouse keratinocytes (m308k cell line) upon TRPV3 activation. We investigated whether this also applies to primary keratinocytes. In line with the previous results, primary keratinocytes from WT mice close a defined 500-*μ*m gap statistically significantly faster than cells from TRPV3 KO mice (Fig. 1, A and B). After 18 hours, WT keratinocytes closed 51.1% ± 4.6% of the gap, whereas the TRPV3 KO cells closed 36.6% ± 3.7% (Fig. 1B).

### 3.2 Primary WT keratinocytes display TRPV3-mediated Ca^2+^ sparklets

**Fig. 2:**
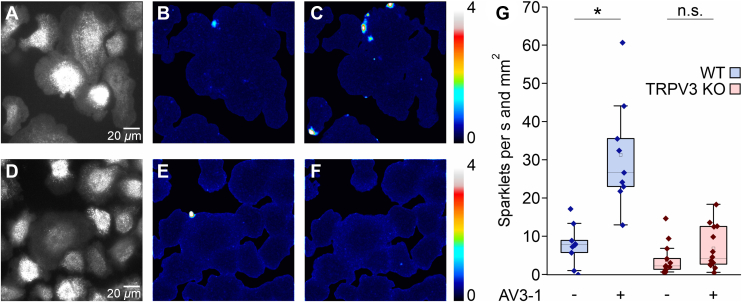
AV3-1-induces Ca^2+^ sparklets in WT keratinocytes. (A, D) WT (A) and TRPV3 KO (D) keratinocytes were loaded with the low-affinity Ca^2+^ indicator Cal520ff, and imaged by gap-free TIRF microscopy at 20 frames per second. (B, C, E, F) Maximum projection of the variance analysis of the corresponding raw data shown in (A) and (D) within 50 seconds before AV3-1 addition (B, E) and within 50 seconds after AV3-1 (3 *μ*M) addition (C, F). Note the preferred localization of Ca^2+^ sparklets in the leading edges of WT keratinocytes (C). The intensity variance was calculated over consecutive bins of 10 frames and divided by the respective mean fluorescence to obtain a measure of local Ca^2+^ fluctuations as represented by a pseudocolor scale. The maxima of 100 bins before and after AV3-1 addition, respectively, are projected in each image. (G) Quantitative analysis of the appearing sparklets per second and mm^2^ cell area in WT (blue boxes) and TRPV3 KO (red boxes) keratinocytes (n_WT_ = 9, n_TRPV3 KO_ = 14, ∗*P* ≤ .05, data not normally distributed). n.s., statistically not significant.

According to literature, cell migration is steered by localized, short-living Ca^2+^ signals (Ca^2+^ sparklets) rather than global (Ca^2+^)_i_ increases.^23^ To elucidate whether TRPV3 plays a role in subcellular Ca^2+^ signaling, we imaged primary keratinocytes with TIRFM. WT cells loaded with the low-affinity Ca^2+^ indicator Cal520ff (K_D_ = 9.8 *μ*M) responded with a statistically significant increase in subplasmalemmal Ca^2+^ sparklets upon addition of 3 *μ*M AV3-1 (Fig. 2). TRPV3-mediated Ca^2+^ sparklets occurred mostly in the leading edges of the cells, supporting the notion of Ca^2+^ sparklet-steered migration (Fig. 2C, Supplemental Movie). TRPV3 KO–derived keratinocytes exhibited no statistically significant increase of Ca^2+^ sparklets upon AV3-1 (3 *μ*M) addition. Hence, the signals depict TRPV3-specific Ca^2+^ influx.

### 3.3 TRPV3 activation stimulates phosphoinositide 3-kinase

**Fig. 3:**
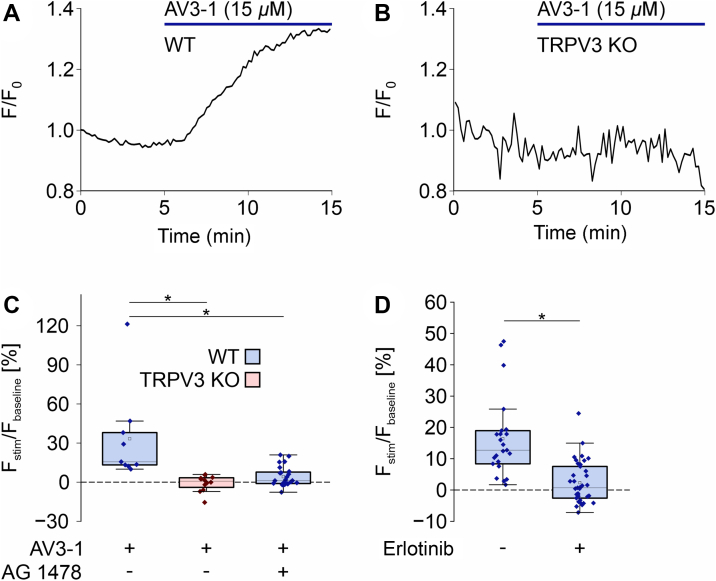
TRPV3 activation stimulates PI3 kinases in an EGFR-dependent manner. (A, B) TIRF microscopy signals of WT (A) and TRPV3 KO (B) keratinocytes transfected with 4 *μ*L LipofectAMINE 2000 and 1 *μ*g cDNA encoding YFP-GRP1(PH), a translocating biosensor of phosphatidylinositol (3,4,5)-trisphosphate formation. Blue bars indicate AV3-1 addition (15 *μ*M). (C) Statistical analysis of the TIRFM signals evoked by AV3-1, as shown in (A) and (B) in WT (blue boxes) and TRPV3 KO (red boxes) keratinocytes. EGFR in WT cells was inhibited by AG 1478 (100 nM) in a second set of experiments (n_WT_ = 9, n_KO_ = 12, n_AG 1478_ = 24, data not normally distributed). (D) WT keratinocytes were transfected with 4 *μ*L LipofectAMINE 3000 and 1 *μ*g cDNA encoding YFP-GRP1(PH) in a third set of experiments. The graph analyses the statistical fluorescence increase caused by the addition of AV3-1 (15 *μ*M) without and with prior inhibition of EGFR by erlotinib (10 *μ*M, n_control_ = 23, n_erlotinib_ = 36, ∗*P* ≤ .05, data not normally distributed).

Because TRPV3 opening has been described to activate the EGFR^9^ and the phosphoinositide 3-kinase (PI3K) is a known downstream target of EGFR mediating cell migration,^24^ we investigated a possible PI3K activation downstream of TRPV3 channel opening. To this end, AV3-1 (15 *μ*M) was applied to YFP-GRP1(PH)-expressing primary keratinocytes to induce a global (Ca^2+^)_i_ increase. GRP1(PH) acts as a sensor for PI3K activity, because it translocates to the plasma membrane upon PI3K-mediated formation of phosphatidylinositol (3,4,5)-trisphosphate in the lipid bilayer.^25^ Transfected WT keratinocytes showed a statistically significant increase in the fluorescence upon AV3-1 addition (Fig. 3A), whereas transfected TRPV3 KO keratinocytes displayed no visible response to the compound (Fig. 3B). To elucidate whether the observed TRPV3-dependent PI3K activation is EGFR-mediated, the EGFR inhibitors AG 1478 (100 nM) or erlotinib (10 µM) were applied onto WT keratinocytes in a second and third set of experiments. Both EGFR inhibitors completely abolished the response of WT cells to AV3-1, confirming the signal transduction from TRPV3 channels via EGFR to PI3K (Fig. 3C). Sufficient EGFR inhibition by AG 1478 was tested by adding EGF (10 ng/mL) to HEK cells expressing YFP-GRP1(PH) in the absence and presence of AG 1478 (Supplemental Fig. 2). The applied AG 1478 concentration (100 nM) statistically significantly inhibited the translocation of the fluorescent biosensor, ensuring adequate EGFR inhibition.

### 3.4 WT and TRPV3 KO mice display no difference in skin wound healing

**Fig. 4:**
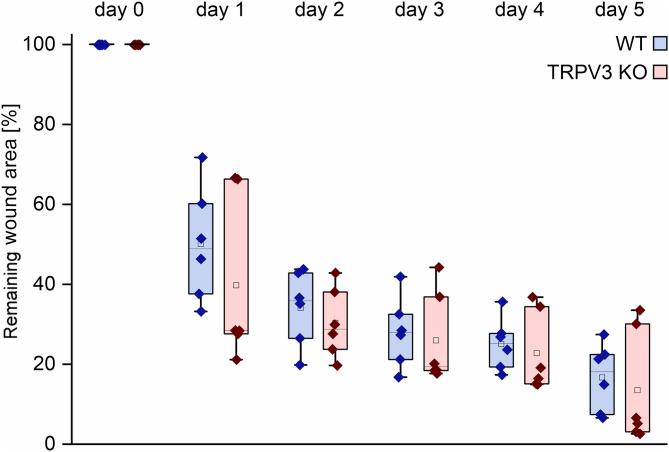
WT and TRPV3 KO mice display no statistically significant difference in dermal wound areas over 5 days in vivo. Remaining wound areas in WT (blue boxes) and TRPV3 KO (red boxes) mice, at the indicated number of days after biopsy. On day 0, two 5-mm biopsy punch wounds were excised on the dorsal skin of the mice, equally distanced on each side of the midline (n_WT_ = 6, n_TRPV3__KO_ = 6, data not normally distributed).

The described in vitro results, in combination with the literature, may suggest an accelerating effect of TRPV3 activation on dermal wound healing in vivo. To test this hypothesis, we excised two 5-mm biopsy punch wounds on the back of WT and TRPV3 KO mice and investigated the wound area over 5 consecutive days of healing. Surprisingly, no statistically significant difference in the wound closure of WT and TRPV3 KO mice could be observed (Fig. 4), thereby confirming earlier observations by Miyamoto et al.^15^ Note that a slight wound expansion in the first 2 days postsurgery is not uncommon (Fig. 4).^26^

### 3.5 TRPV3 activation does not accelerate wound closure in vivo

Next to the comparison of WT and TRPV3 KO wounds, a potential accelerating effect of AV3-1 on dermal wound healing in vivo was investigated. To this end, AV3-1 (100 *μ*M) was applied on one of the 5-mm punch wounds, whereas the contralateral wound received the corresponding vehicle. The administered concentration of 100 *μ*M AV3-1 was determined in a preliminary dose-escalation scheme (data not shown), where 1, 10, and 100 *μ*M AV3-1 were tested, based on the in vitro EC_50_ = 1.45 ± 0.26 *μ*M of AV3-1 (J. Janenz et al, manuscript in preparation). Because 100 *μ*M AV3-1 first showed promising results in contrast to 10 *μ*M AV3-1, the higher concentration was used for the following experiments.

**Fig. 5:**
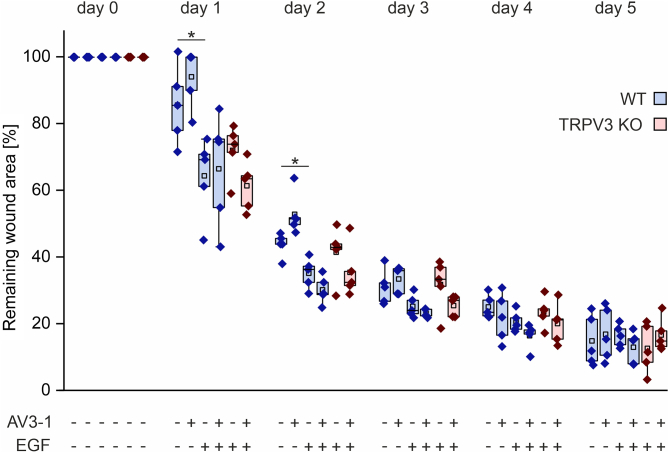
Pharmacological activation of TRPV3 does not induce a statistically significant acceleration of dermal wound healing with or without topical application of EGF. Remaining wound areas in WT (blue boxes) and TRPV3 KO (red boxes) animals over 5 days are shown. Mice were either treated with AV3-1 (100 *μ*M) or the combination of AV3-1 (100 *μ*M) and EGF (10 ng/mL). The treatments were applied on one of the 5-mm biopsy punch wounds, whereas the contralateral wound received the corresponding vehicle or EGF alone (10 ng/mL) (*n* = 5 for each group, ∗*P* ≤ .05, data not normally distributed).

In line with the results described earlier (Fig. 4), AV3-1 did not induce a statistically significant difference between the areas of AV3-1-treated and vehicle-treated WT wounds (Fig. 5). Because local degradation of topically applied AV3-1 by components of the wound exudate may have caused loss of biological activity, we analyzed the stability of the compound in serum, which is a major source of wound exudate. To this end, AV3-1 was incubated in vitro in human serum at 34 °C for 0, 6, or 24 hours, and the ability to activate TRPV3 was assessed in a functional multiwell Ca^2+^ assay, applying TRPV3-expressing HEK cells as a biosensor of AV3-1 activity. There was no statistically significant decrease in the AV3-1-induced TRPV3 activation after the different incubation times (Supplemental Fig. 3). We, therefore, conclude that the compound is not strongly degraded by serum components.

According to Maier et al^6^ the combination of EGF with a TRPV3 activator accelerates keratinocyte migration in a superadditive manner. Therefore, both compounds AV3-1 (100 *μ*M) and EGF (10 ng/mL) were applied on 1 wound and EGF alone (10 ng/mL) on the corresponding contralateral wound. Unexpectedly, the compound combination also failed to accelerate the dermal wound healing (Fig. 5). EGF induced an acceleration of wound closure on days 1 and 2 after wounding, but AV3-1 could not further enhance this effect. The wound areas of treated WT and TRPV3 KO wounds exhibited no significant difference (Fig. 5), confirming the results of non-treated WT and TRPV3 KO mice (Fig. 4).

### 3.6 AV3-1 treatment does not increase the histologically determined reepithelialization

**Fig. 6:**
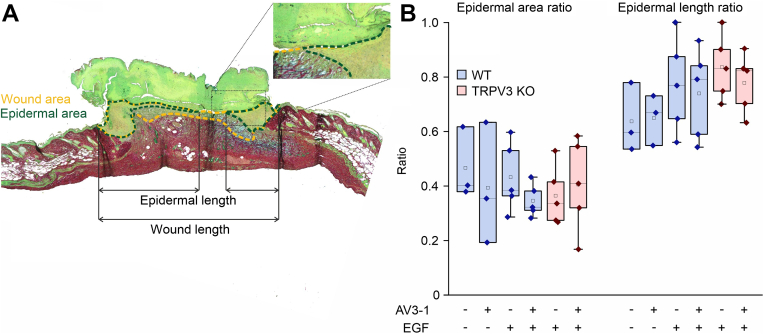
AV3-1 treatment with or without co-application of EGF causes no statistically significant increase in the extent of reepithelialization. (A) RGB-stained slice of a wound on day 5 postsurgery with the following analyzed wound parameters: wound area (orange dotted line), epidermal area (green dotted line), and wound length and epidermal length (indicated by arrows). The insert shows the boundary between the scab and epidermis. (B) Statistical analysis of the epidermal area ratio (epidermal area/wound area) and the epidermal length ratio (epidermal length/wound length) of WT (blue boxes) and TRPV3 KO (red boxes) wounds as shown exemplary in (A) (n_WT, DMSO_ = 3, n_WT, AV3-1_ = 3, n_WT, EGF_ = 5, n_WT, AV3-1, EGF_ = 5, n_TRPV3 KO, EGF_ = 5, n_TRPV3 KO, AV3-1, EGF_ = 5, data normally distributed).

The AV3-1-treated and non-treated wounds were further analyzed histologically to evaluate a possible TRPV3 effect on a more detailed level (Fig. 6). To assess the degree of reepithelialization, the percentages of the epidermal area and epidermal length in the total wound bed were calculated (Fig. 6A). The wound area includes light green colored keratinocytes and blue stained granulation tissue (Fig. 6A, orange dotted line), whereas the epidermal area only includes the keratinocyte covered regions (Fig. 6A, green dotted line). The bright green–stained scab was excluded from the analyses. Unexpectedly, the treatments could not increase the degrees of reepithelialization compared to the vehicle treatment, supporting the macroscopic results. Neither the proportion of epidermal area, which is mainly defined by keratinocyte proliferation, nor the keratinocyte migration–dependent epidermal length proportion differed between AV3-1-treated and vehicle-treated wounds with or without co-application of EGF (Fig. 6B). Treated WT and TRPV3 KO wounds exhibited similar skin anatomies without any visible differences in the epidermal structure (data not shown).

## 4 Discussion

In this study, we investigated the in vitro and in vivo effects of the Ca^2+^-permeable TRPV3 channel on mouse keratinocytes and skin wound healing. TRPV3 KO keratinocytes showed a statistically significantly slower gap closure than primary WT keratinocytes in vitro (Fig. 1). The accelerating role of TRPV3 activity in cell migration and proliferation in vitro is well known.^27^^,^^28^ Cells of the mouse keratinocyte cell line m308k migrated statistically significantly faster after TRPV3 activation, and the effect was superadditively stimulated by the combination of a TRPV3 activator and EGF.^6^ TRPV3 and EGFR form a spatial and functional signaling complex,^8^ mediating the accelerated cell migration and proliferation upon TRPV3 activation.^9^^,^^28^^,^^29^ Because of the spatial coupling of TRPV3 channels and EGFR, localized Ca^2+^ influx through TRPV3 could stimulate EGFR activation in a spatially and temporally limited manner. According to the literature, localized, short-lived Ca^2+^ flickers steer cell migration.^23^ In primary WT keratinocytes, such Ca^2+^ sparklets could be observed after addition of the TRPV3-specific activator AV3-1, but were absent in TRPV3 KO keratinocytes. Of note, the vast majority of these Ca^2+^ sparklets were located in the leading edges of WT keratinocytes (Fig. 2), suggesting a role of localized, short-lived TRPV3 openings in the steering of mouse keratinocyte migration in a 2D cell culture setting. According to Maier et al,^6^ a heterologously expressed fluorescent TRPV3 fusion protein is evenly distributed in the plasma membrane of immortalized m308 keratinocytes with no enhancement at cell margins or in leading edges. A likely reason for the dominant appearance of Ca^2+^ sparklets along the cell edges may be the flat geometry of the leading edges, restricting the diffusion of influxing Ca^2+^ ions out of the subplasmalemmal space that is preferentially imaged in TIRFM.

The PI3 kinase has been described to act in the migration, mediating the downstream cascade of EGFR.^24^ In primary WT keratinocytes, we could stimulate PI3K by activating TRPV3 with AV3-1 (Fig. 3, A–C). The signal was absent in TRPV3 KO cells and sensitive to the EGFR inhibitor AG 1478, which points to a PI3K stimulation downstream of EGFR transactivation by TRPV3 opening.^9^

Based on the extensive in vitro findings on TRPV3 accelerating keratinocyte migration and proliferation, the suggested role of the channel as a promising target for improving wound healing seems logical.^13^ Therefore, in vivo studies of TRPV3’s effect on dermal wound healing are of particular interest. Unexpectedly, TRPV3 did not exhibit any accelerating effect on dermal wound healing in vivo (Figs. 4 and 5) in the used well established wound healing model.^1^^,^^15^^,^^30^ Neither the comparison of WT with TRPV3 KO wound areas nor of AV3-1-treated with vehicle-treated wound areas yielded a statistically significant difference. These findings align with the results of Miyamoto et al,^15^ who studied the in vivo wound healing kinetics of WT and TRPV3 KO mice on a 129S1/SvImJ background. Background-specific differences are described in the literature regarding the temperature sensitivity and skin anatomy of WT and TRPV3 KO mice.^8^^,^^15^^,^^31^ Therefore, a potential influence of the genetic background on the role of TRPV3 in dermal wound healing seemed possible, but could not be confirmed with the used C57BL/6J WT and genetically matching TRPV3 KO model. Supporting the notion of a minor role of TRPV3 in dermal wound healing, the topical application of TRPV3 activator AV3-1 with or without EGF could not induce an acceleration of the wound closure compared to the vehicle control (Fig. 5). Possibly, higher animal numbers might lead to a statistically significant TRPV3-dependent increase in the healing rates, but if so, the effect size would remain small and the biological relevance of this effect would still be questionable. EGF increased the wound healing speed, independent of AV3-1, at days 1 and 2 after wounding, coinciding well with the described enhanced EGFR expression in the first 2 days after dermal wounding.^32^

One reason for a missing TRPV3 impact on dermal wound healing in vivo might be that the global TRPV3 KO caused the development of compensatory mechanisms to bypass the lack of TRPV3. Additionally, a potential toxicity of AV3-1 could delay the wound healing in the in vivo model, although no relevant toxic effects of AV3-1 were observed in cell culture experiments (J. Janenz et al, manuscript in preparation). An insufficient stability of AV3-1 in the wound bed seems unlikely because the biological ability to activate TRPV3 was retained even after incubating the compound for 24 hours at 34 °C in human serum (see Supplemental Fig. 3).

Another reason for the lack of TRPV3 effect could be a more important role of transient receptor potential vanilloid 4 (TRPV4). Taivanbat et al^33^ observed a statistically significant slower skin wound healing in TRPV4 KO mice on days 2–6 after wounding in a comparable model. In the wound bed, the endogenous TRPV4 activator arachidonic acid is released,^34^ whereas for TRPV3, such an activator is missing. In contrast, in the oral cavity, Aijima et al^14^ actually identified a decelerated wound healing in TRPV3 KO mice after molar extraction, whereas TRPV4 KO did not exhibit any statistically significant influence on oral wound closure. Future studies will be necessary to distinguish between the distinct roles of TRPV4 and TRPV3 in the wound healing of different epithelial tissues. An additional reason for the lack of effect of TRPV3 activation on dermal wound healing might be the chosen full-thickness wound model. Recent studies showed a statistically significantly increased keratinocyte proliferation in vivo upon TRPV3 activation using healthy skin^35^ and a tape-stripping model without disruption of the dermis.^36^ Therefore, the infliction of non-full-thickness wounds may be beneficial to investigate TRPV3’s role in skin barrier formation in vivo.

The present study observed no statistically significant effect of TRPV3 and its activator AV3-1 on the closure and reepithelialization of dermal excisional wounds in mice in vivo, which aligns well with the findings of Miyamoto et al.^15^ Therefore, the described in vitro results cannot be directly translated into an in vivo setting. Although future studies with different models and investigation parameters may still identify an influence of the channel on dermal wound healing, TRPV3 might not be the promising wound healing target as hoped before.

## Conflict of interest

All authors declare no conflicts of interest.
