# Supplementary material for: Transient receptor potential vanilloid 3 activation accelerates keratinocyte migration in vitro but not dermal wound healing in vivo
Source: Mol Pharmacol. 2025 Oct 27;107(12):100084. doi: 10.1016/j.molpha.2025.100084 (PMC12799580; doi:10.1016/j.molpha.2025.100084)
Supplement: Supplementary Material [file mmc1.docx]

**Transient receptor potential vanilloid 3 activation accelerates keratinocyte migration in vitro but not dermal wound healing in vivo**

Carolin Zosel ^a^, Anne-Kathrin Krause ^a^, Anne Müglitz ^a^, Yan-Qin Zuo ^a^, Ute Krügel ^a^, Michael Schaefer ^a^

^a^ Rudolf-Boehm-Institute for Pharmacology and Toxicology, Leipzig University, Härtelstrasse 16-18, 04107 Leipzig, Germany


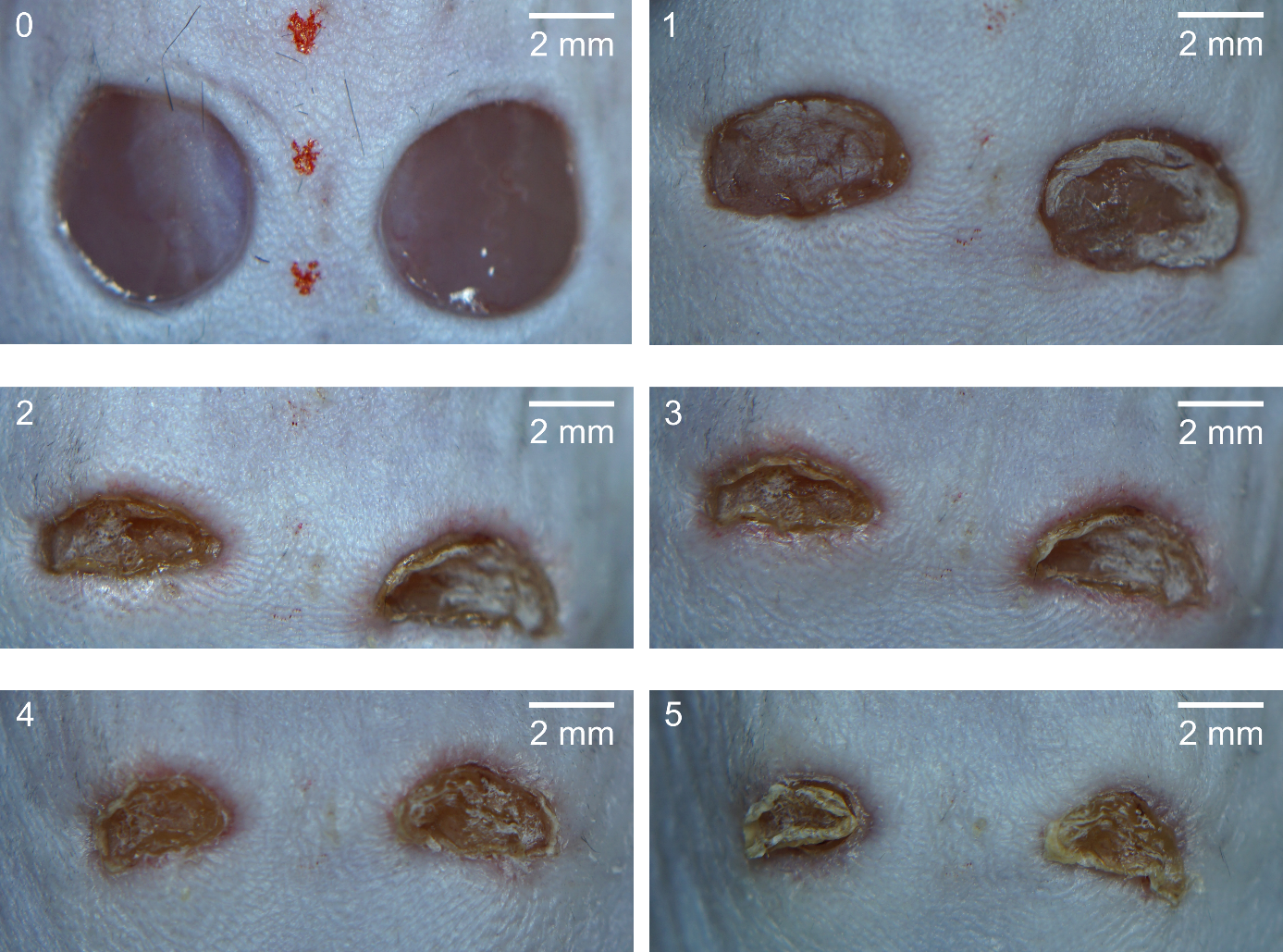


**Supplemental Figure 1.** Depiction of the *in vivo* wound healing assay

Two 5-mm biopsy punch wounds were excised on the dorsal skin of mice and photographs were taken directly thereafter (day 0, upper left panel) and daily for the next five days (remaining panels, labeled with days after biopsy). The equally sized wounds were located on each side of the dorsal midline. One randomly assigned wound received treatment and the contralateral wound the corresponding vehicle. Wound areas were analyzed using ImageJ.


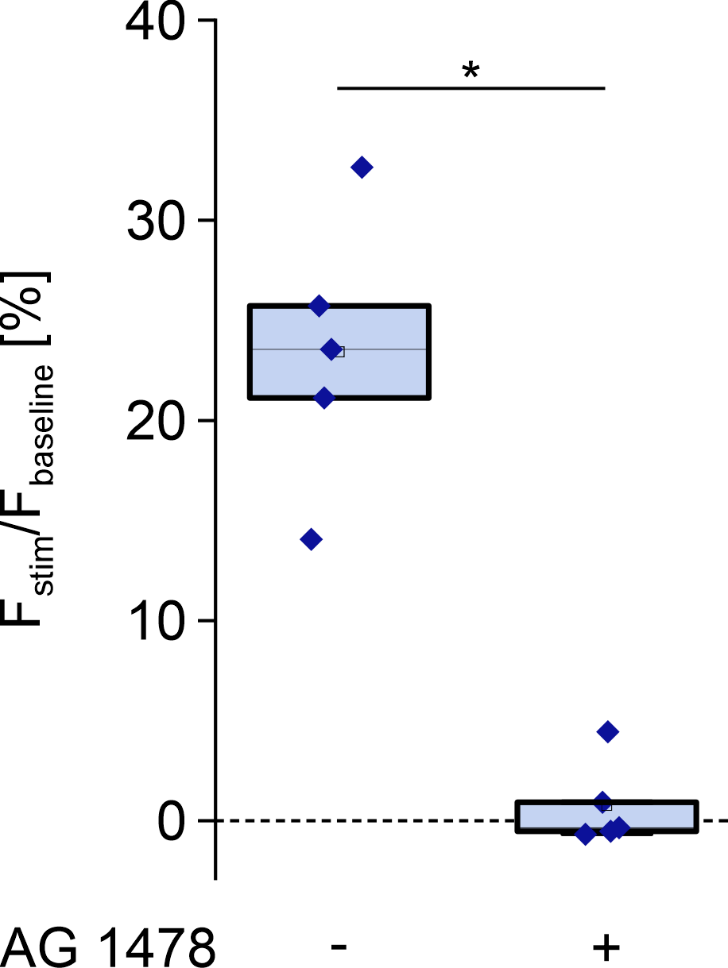


**Supplemental Figure 2.** TIRF microscopy measurements of HEK cells with and without AG 1478

HEK cells stably expressing mouse TRPV3 were transfected with 4 µl jetPEI and 1 µg of cDNA encoding YFP-GRP1(PH), a translocating biosensor for PI3 kinase activity. Depicted is the fluorescence increase caused by the addition of EGF (10 ng/ml) in the absence and presence of the EGFR inhibitor AG 1478 (100 nM, n = 5 per group, * P ≤ 0.05, data not normally distributed).


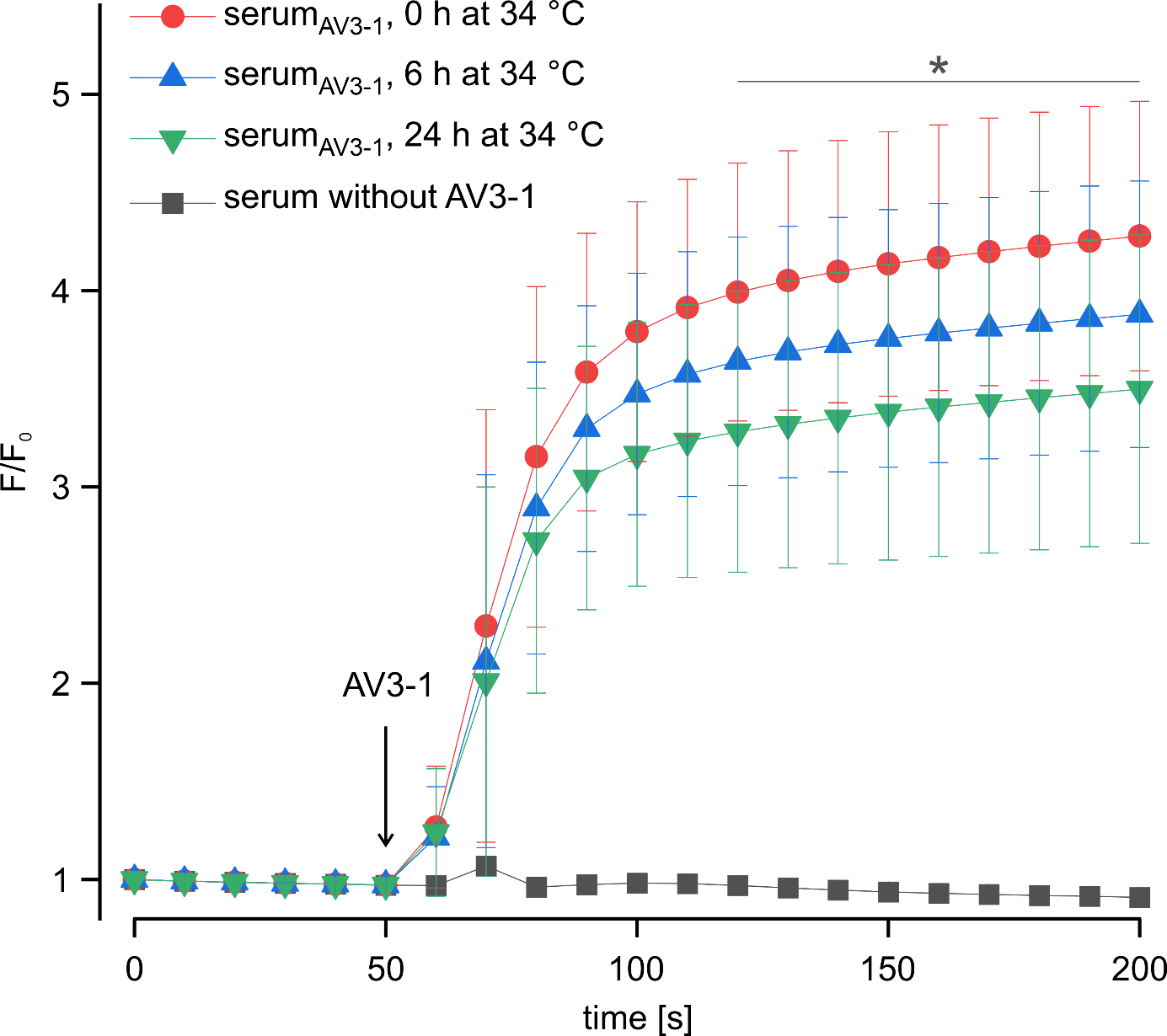


**Supplemental Figure 3.** AV3-1 reliably activates TRPV3 channels after incubation of the compound in serum

AV3-1 (10 mM) was incubated for 0 h (red circles), 6 h (blue triangles) or 24 h (green triangles) at 34 °C in serum. After incubation, samples were diluted in HBS buffer, and a final concentration of 10 µM AV3-1 + 0.1% serum was applied at the indicated time point (arrow) to TRPV3-expressing HEK cells loaded with the Ca^2+^ indicator Fluo-4. As control, 0.1% serum without added AV3-1 (grey squares) was used. Statistical significance was reached between the AV3-1 containing samples and the AV3-1-free serum control at the indicated time points (n_serum AV3-1 free_ = 3, n_AV3-1,0 h_ = 6, n_AV3-1,6 h_ = 6, n_AV3-1,24 h_ = 6, * P ≤ 0.05, data normally distributed, mean ± S.D.).

**Supplemental Movie.** AV3-1-induced Ca^2+^ sparklets mainly occur in the leading edges of WT keratinocytes

Variance analysis movie of WT keratinocytes stimulated with AV3-1 (3 µM) and imaged with 20 frames s^-1^. Variance was calculated over consecutive bins of 10 frames each and divided by the mean fluorescence. The variance analysis movie depicts Ca^2+^ sparklets ten times faster than the raw fluorescence data.
